# Mapping end-of-life care for patients with neurological conditions in German hospices: a point prevalence survey

**DOI:** 10.1136/bmjno-2025-001404

**Published:** 2026-02-25

**Authors:** Sarah Kristina Bublitz, Stefan Lorenzl, André Klima, Susann Schmidt, Benno Schäffer, Sabine Gleich

**Affiliations:** 1 Institute of Palliative Care, Paracelsus Medical Private University, Salzburg, Salzburg, Austria; 2 Neurology and Palliative Care, Krankenhaus Agatharied GmbH, Hausham, Bavaria, Germany; 3 Gesundheitsreferat, Landeshauptstadt München, Munich, Germany; 4 Institut für Rechtsmedizin, Ludwig-Maximilians-Universität München, Munich, Germany

**Keywords:** PARKINSON'S DISEASE, ALS, MOTOR NEURON DISEASE

## Abstract

**Background:**

Access to palliative care for patients with neurological diseases remains limited. Contributing factors include difficulties in predicting disease trajectories, resource constraints in long-term care and challenges in identifying the end-of-life phase—often compounded by communication and cognitive impairments.

**Methods:**

We conducted a national point-prevalence survey among German inpatient hospices using an online questionnaire.

**Results:**

The response rate was 44%, with 83% of participating hospices providing complete datasets. Most patients in hospices suffered from oncological diseases (n=785; 77.3%), including primary brain tumours (n=102; 10.0%). At the time of the survey, neurological diagnoses accounted for approximately 5% of hospice admissions. While 51% of hospices reported having access to neurological consultation, this was usually informal or ad hoc. 19% reported no current access to a neurologist but considered such collaboration desirable.

**Conclusions:**

This survey provides an overview of the current representation of patients with neurological conditions in German inpatient hospices. The findings reveal limited structured collaboration between neurology and palliative care, alongside structural and societal barriers that complicate timely hospice referral and end-of-life planning. Strengthening interdisciplinary cooperation, enhancing neurologists’ engagement in palliative care and expanding specialised outpatient support for patients and families are essential to improving equitable and needs-based end-of-life care for individuals with neurological conditions.

**WHAT IS ALREADY KNOWN ON THIS TOPIC**

- Neuropalliative care is recognised as an essential, multidimensional approach for patients with neurological diseases. However, access remains limited due to challenges in predicting disease trajectories, restricted resources and gaps in mutual understanding between neurology and palliative care. Previous research demonstrates broad agreement on the benefits of such integrated care but also emphasises the persistent need for closer interdisciplinary collaboration.

**WHAT THIS STUDY ADDS**

- This study provides representative point prevalence data on inpatient hospice utilisation among individuals with neurological conditions in Germany. By showing that patients with neurological conditions constitute about 5% of admissions compared with 87% with oncological diagnoses, and revealing the limited interest in neurologist consultations within hospices, our findings highlight a substantial deficit in end-of-life care and cross-specialty cooperation. These results provide essential evidence for shaping targeted national strategies to integrate palliative expertise into neurology and strengthen neurological perspectives within palliative care.

**HOW THIS STUDY MIGHT AFFECT RESEARCH, PRACTICE OR POLICY**

- This study underscores the need to intensify interprofessional education and establish low-barrier referral pathways. Furthermore, the findings will stimulate research to more closely examine the specific support requirements of patients with neurological conditions (such as Parkinson’s disease) and their caregivers, informing the development of future sustainable care models.

## Introduction

 Neuropalliative care is an emerging field that addresses the palliative needs of individuals with neurological diseases. Drawing from the ‘total pain’ model, this specialised care encompasses the physical, emotional, spiritual, practical and social dimensions of patient needs.^1^ However, access to palliative care remains limited for rapidly progressive neurological diseases, like amyotrophic lateral sclerosis (ALS) and glioblastoma, and even more so for slower progressive diseases, such as Parkinson’s disease and other movement disorders.^2^ Contributing factors include the inherent challenges in predicting disease trajectories, resource limitations within long-term care and the complexities of identifying the end-of-life phase, further compounded by communicative and cognitive impairments.

A survey of neurologists and palliative care physicians revealed a strong consensus on the benefits of palliative care in enhancing quality of life, bolstering caregiver support, improving psychological well-being and facilitating complex decision-making.^2^ Nevertheless, only 18% of palliative care professionals considered their knowledge of neurology to be expert or very good, while 16% of neurologists described their palliative care expertise likewise. This highlights a critical, but currently limited, need for collaboration between these essential specialties.

Cross-regional and health system comparisons of end-of-life care are complicated by variations in terminology. In some countries, the meaning of ‘hospice care’ relates to a philosophy of care rather than a type of care setting, and it might be used more or less synonymously with the term ‘palliative care’.^3^ In Germany, ‘hospice’ specifically denotes nurse-led residential or inpatient facilities. These facilities provide comprehensive, multidimensional end-of-life care for individuals with terminal illnesses whose complex symptoms necessitate specialised support outside of their homes, with coverage typically extended by health insurance for up to a maximum of 6 months according to the Statutory Health Insurance Framework Agreement.^4^ Beyond inpatient hospices, palliative care units within hospitals primarily focus on crisis intervention and medical stabilisation.

Given these challenges and definitional nuances, this study aims to document current access to inpatient hospice care for individuals with neurological diseases in Germany via a point prevalence survey. We also explored inpatient hospices’ capacity for neurologist consultations (and the desire for such collaboration), as well as the availability and interest in telemedicine support.

## Methods

The point prevalence survey was conducted as an online survey. A draft version of the questionnaire was produced and repeatedly discussed for revisions between the authors. The survey was developed using LimeSurvey in accordance with the Checklist for Reporting Results of Internet E-Surveys.^5^

The survey consisted of a total of 13 items grouped into four sections: four items on hospice features, five items on patient characteristics and four items on the possibilities of obtaining external consultations (*for the full survey, see the* online supplemental documents). Usability and technical functionality of the electronic questionnaire were tested by the authors before fielding the questionnaire.

A complete list of contact addresses for inpatient hospices was compiled via the hospice and palliative care guide of the German Society for Palliative Medicine (Deutsche Gesellschaft für Palliativmedizin).^6^ Prior to sending the invitation link, all hospice federations in the German federal states were informed about the study to encourage participation. Subsequently, all 264 hospices in Germany were contacted via email, which included an invitation letter outlining the aims of the survey, its anonymity and an expected completion time of 10–15 min, along with a link and quick response code to the web survey. No incentives were offered to participants for taking the time to fill out the survey. The survey was initiated on 22 January 2025.

Data analysis was performed using Microsoft Excel.

## Results

### Response rate and information on inpatient hospices in Germany

Between 22 January 2025 and 28 April 2025, responses were received from 124 inpatient hospices in 14 of the 16 German federal states (participation rate 44%). Table 1 shows the number of hospices per state, according to the German Society for Palliative Medicine,^6^ and the participation rates in the online survey per state. In total, data were retrieved from 1341 hospice beds, representing 46% of all residential hospice beds in Germany. Of these 1341 beds, 1261 beds were occupied at the time of the survey (94% occupancy rate).

**Table 1:** Current number of hospices and hospice beds in German federal states and responses to our survey

Federal state	Number of hospices (percentage of all hospices)	Number of hospice beds (percentage of all German hospice beds)	Number of hospices taking part in the survey (percentage of all participating hospices)	Number of hospice beds in the survey	Number of occupied beds at the time of the survey
Baden-Württemberg	41 (14.4)	350 (11.9)	17 (13.7)	138	130
Bavaria	20 (7.0)	207 (7.0)	19 (15.3)	210	199
Berlin	17 (6.0)	241 (8.2)	10 (8.1)	139	134
Brandenburg	13 (4.6)	146 (5.0)	4 (3.2)	54	47
Bremen	3 (1.1)	24 (0.8)	0	0	0
Hamburg	6 (2.1)	89 (3.0)	5 (4.0)	68	66
Hesse	23 (8.1)	233 (7.9)	5 (4.0)	49	46
Lower Saxony	20 (7.0)	193 (6.6)	10 (8.1)	101	94
Mecklenburg-Western Pomerania	7 (2.5)	70 (2.4)	1 (0.8)	10	5
North Rhine-Westphalia	72 (25.4)	722 (24.5)	28 (23.0)	301	296
Rhineland-Palatinate	18 (6.3)	164 (5.6)	8 (6.5)	79	71
Saarland	4 (1.4)	48 (1.6)	0	0	0
Saxony	12 (4.2)	140 (4.8)	7 (5.6)	80	69
Saxony-Anhalt	6 (2.1)	64 (2.2)	2 (1.6)	26	25
Schleswig-Holstein	11 (3.9)	133 (4.5)	5 (4.0)	54	50
Thuringia	11 (3.9)	119 (4.0)	2 (1.6)	22	19
Without information on federal state			1 (0.8)	10	10
Total	284	2943	124	1341	1261

Of the 124 submitted questionnaires, 109 (88%) were completed by the hospice manager, head of nursing or other administrative employees, 7 (6%) by a nurse and 9 (7%) by a member of the social services team.

Out of the hospices that responded to our survey, 101 provided full datasets (completion rate 83%), sharing information on their patients’ diagnoses. One dataset with data on 10 patients was excluded from the analysis because the data seemed implausible, as the sum of individual diagnoses clearly exceeded the total number of patients. The comprehensive data set covers 1081 hospice beds, representing 37% of all residential hospice beds in Germany, and is the basis of the following descriptive analysis of patient characteristics.

### Patient characteristics

**Figure 1 F1:**
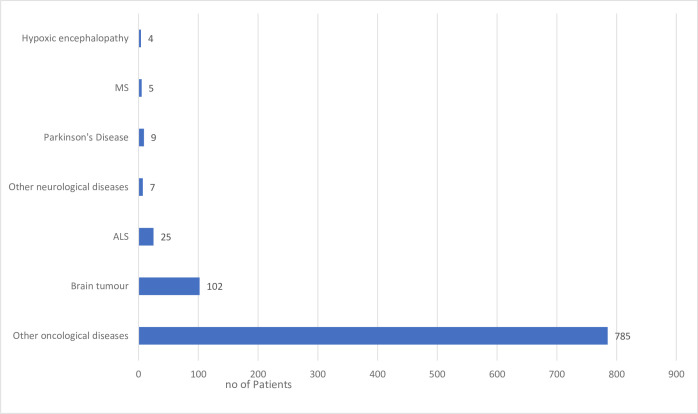
Patients’ diagnoses in German hospices according to our survey. Total number of patients: 1015. ALS, amyotrophic lateral sclerosis; MS, multiple sclerosis.

The collected data reveal that the vast majority of patients in German inpatient hospices in our survey (total number of patients: 1015) are suffering from oncological diseases (n=887, 87.4%), 10.0% of whom suffer from primary brain tumours (n=102) and 77.3% from other oncological diseases (n=785) (*see[Fig F1]* figure 1). At the time of the survey, 25 (2.5%) patients with ALS, 9 (0.9%) with Parkinson’s disease or atypical Parkinson’s syndromes, 5 (0.5%) with multiple sclerosis (MS) and 4 (0.4%) with hypoxic encephalopathy were admitted to inpatient hospices in Germany. Other neurological diagnoses (n=7, 0.7%) that were mentioned as a commentary included dementia, schizophrenia, spinocerebellar ataxia, Huntington’s disease and severe mitochondriopathy. Neurological diagnoses account for 5% of hospice admissions.

### Availability of and interest in external consultations

**Figure 2:**
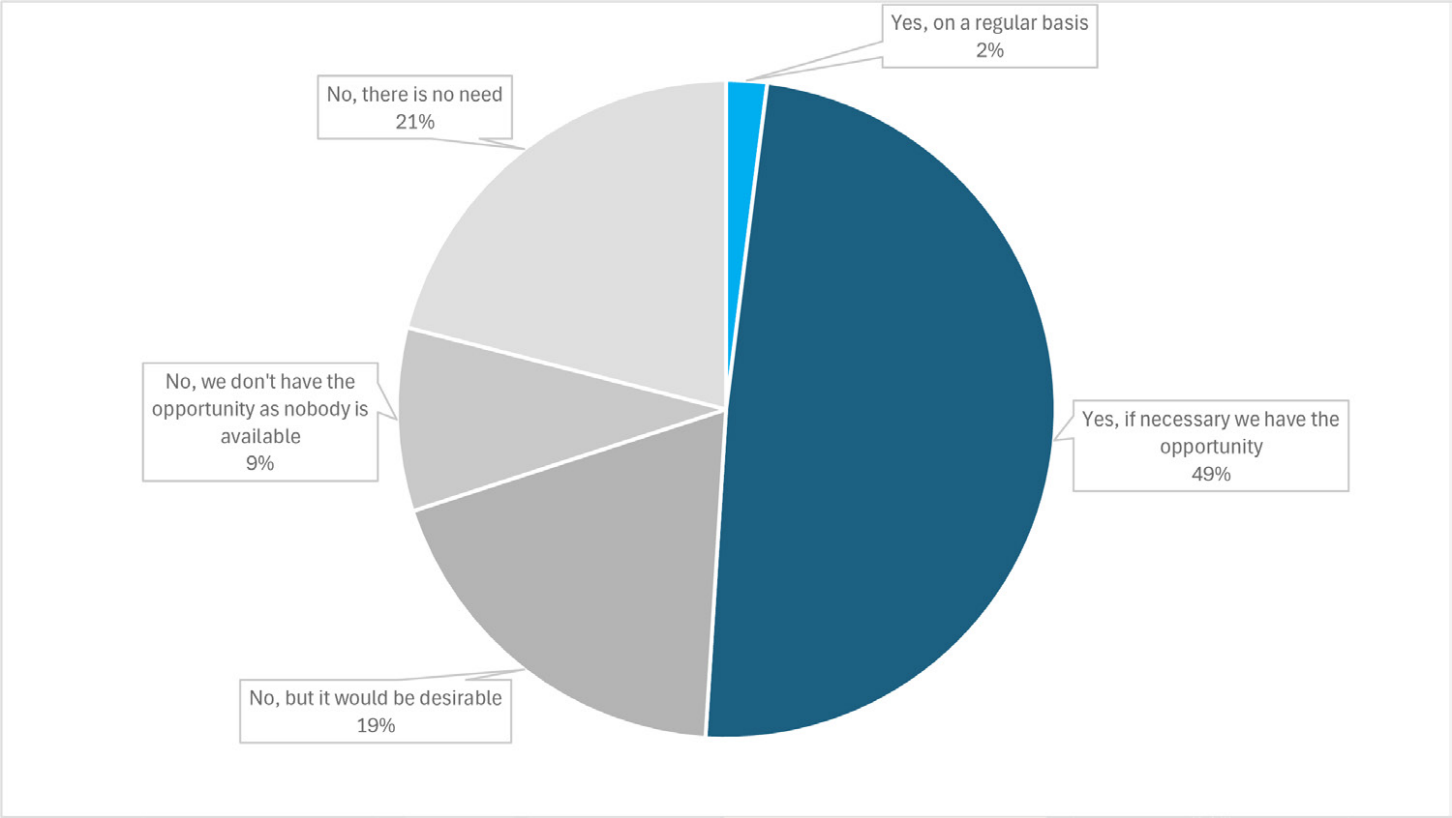
Consultation by neurologists in German hospices according to our survey (n=100).

100 hospices answered the question regarding the option to consult a neurologist. While 51% of inpatient hospices reported having some option to consult a neurologist, most indicated this was an informal ad hoc arrangement rather than a formal collaboration. 19% stated that they currently lack neurologist access but would see this as a desirable goal (*see* figure 2).

Analysis of the commentaries indicates that palliative care physicians who are closely collaborating with hospices often manage neurological symptoms themselves (‘*Due to the close-knit care provided by palliative physicians, co-treatment by other specialists is generally not required in the hospice*’). External support comes through established networks and collaborations (*‘as part of the collaborations within the city’s palliative and hospice network*’), links with palliative care departments in hospitals (*‘The consultant of the palliative care ward, with whom we work closely, also specialises in end-stage neurological diseases*’) or local neurologists (*‘When necessary, we leverage the contact one of our palliative care doctors has with a local neurologist*’). A significant challenge highlighted is the overburdened nature of local neurologists, which makes it difficult for them to provide in-person consultations at hospices (‘*Unfortunately, the local neurologists are so overloaded with work that none of them can come to the hospice; we can only ask for advice by phone*’). In some cases, mixed experiences with previous consultations have clouded trust in local neurologists (‘*Unfamiliar with the emergent symptoms of progressive supranuclear palsy at diagnosis, we received little assistance with adjusting regular and PRN medications*’; PRN means *pro re nat*a, that is, as needed).

Interest in telemedicine consultations was stated by 25% of inpatient hospices, while 75% (74 of 99 answers) showed no interest. Reluctance in applying telemedical support was explained by demographic features (*‘Right now, most guests are over 70 years and use media less*’) or close and sufficient collaboration with palliative care physicians (*‘We are in daily contact, by phone or in person, with the palliative care team, who provide care for almost 100% of our guests*’).

### Relation of potentially eligible patients for hospice and patients in hospice care

To contextualise the proportion of patients receiving hospice care in our point prevalence study, we compared it with the number of potentially eligible patients using publicly available cause-of-death statistics from the German Federal Statistical Office (*see* table 2). Since hospice care is intended for people with life-limiting illnesses in their last months of life, we based our analysis on the assumption that patients who died from a specific diagnosis in a given year would have been eligible for hospice care during their final months of life. This comparison allows us to assess whether the diagnostic mix in our hospice population aligns with the major causes of death at the national level. Our results show that people with malignant brain tumours are over-represented in hospices, while people with Parkinson’s disease and atypical Parkinson’s syndromes are under-represented.

**Table 2:** Relation of the number of potentially eligible patients in hospices according to death statistics from the German Federal Statistical Office and the number of patients currently in hospice

Diagnosis (ICD-10 code)	Number of deaths by diagnosis in Germany in 2023 (according to the German Federal Statistical Office)	Number of patients in hospice (in our point prevalence study)	Relative proportion of patients in hospice in relation to potentially eligible number of patients (%)
ALS (G12.2)	2355	25	1.1
Parkinson syndrome (G20–G23)	14 395	9	0.1
MS (G35)	1694	5	0.3
Malignant brain tumours (C71)	5910	102	1.7
Other oncological diseases (C00–C97 without C71)	224 382	785	0.3

* Total number of deaths in 2023, all diagnoses, according to the German Federal Statistical Office: 1 028 206. Percentage of abovementioned diagnoses in relation to total deaths: 24.2%.

ALS, amyotrophic lateral sclerosis; ICD-10, International Classification of Diseases, Revision 10; MS, multiple sclerosis.

## Discussion

Our findings on the utilisation of inpatient hospices by individuals with neurological conditions in Germany align with and contribute to the broader international discourse on end-of-life care for this patient group. Consistent with data from other countries, our point prevalence study, which represents 37% of all inpatient hospice beds in Germany and is thus considered representative, reveals that neurological diagnoses account for approximately 5% of inpatient hospice admissions, while the vast majority of 87% of patients suffer from oncological diseases (including primary brain tumours).

This disparity indicates gaps in interdisciplinary collaboration and education. The observation that hospices were largely indifferent to neurological consultations may reflect limited experience among neurologists in managing very advanced disease stages and palliative situations. This potential gap is further underscored by the limited representation of neurologists within the German Society for Palliative Medicine, with only 111 neurologists among approximately 3500 physician members (data as of May 2025, personal communication). A recent US study on the collaboration between ALS clinicians and palliative care specialists^7^ draws similar conclusions, emphasising the need to strengthen bi-directional education, specialist palliative services and interdisciplinary collaboration. Notably, 26% of respondents (both ALS and palliative care clinicians) in that study reported referring patients with ALS to hospice later than they otherwise would—or not at all—due to concerns about the quality of care, equipment or services available. In addition, the low representation of patients with neurological conditions in inpatient hospices contrasts with the substantial population-level relevance of neurological diseases at the end of life. In Germany, 53 155 people died from cerebrovascular diseases (ICD-10 I60–I69) and 59 325 from dementia (ICD-10 F00–F03) in 2023, according to the German Federal Statistical Office, placing these conditions among the leading causes of death, particularly in older age groups. Against this background, the small proportion of patients in hospices with neurological diagnoses observed in our study suggests a mismatch between the epidemiological burden of neurological diseases and their visibility within specialist inpatient hospice care.

Beyond the professional gap, several complex factors may contribute to a low utilisation of hospices and palliative care in general by patients with neurological conditions. In most Western countries, there is a general societal preference for dying in one’s usual place of care, often the home. In Germany, about 50% of the population expresses this wish,^8^ yet most deaths still occur in institutional settings,^9^ where the quality of end-of-life care is often perceived as less favourable compared with home-based care. For individuals with long-term neurological conditions, the trajectory of ‘progressive dwindling’, characterised by accumulating disability and frailty, can make home care and dying at home particularly challenging.^10^ Other clinical challenges in the provision of palliative care in neurology include uncertainty regarding prognosis, inconsistencies in information and understanding among patients, caregivers, family and healthcare providers and care provider distress associated with loss of personhood, death and conversations around dying.^11^ In the UK, rates of hospital death for people with long-term neurological conditions are high, particularly for those with Parkinson’s disease (above 40%) and even higher for people with MS (about 55%).^12^ A German study on patients with Parkinson’s disease found that only 5% preferred an inpatient hospice as their place of care and death, underscoring the complexities of patient and caregiver preferences within this population.^13^

Although home care poses considerable challenges for family members, many family members strive to care for their relatives at home, often relying heavily on specialised palliative home care teams. Family caregivers typically provide the majority of care and also shoulder most of the associated costs.^14^ Through their dedicated efforts, these caregivers often gain an expert understanding of the patient’s needs. This expertise can lead to concerns that institutional care may not be able to meet all of the patient’s demands, a fear that has been substantiated by research.^15^ Particularly in Parkinson’s disease, access to palliative care depends on timely information, emotional preparation and support around end-of-life and bereavement.^16^ Identifying appropriate triggers for palliative referral remains difficult, and even close relatives may not recognise the proximity of death, leaving them unprepared for sudden crises.^17^

Furthermore, challenges arise from the discrepancy between the estimated survival prognosis and the duration of hospice stays approved by the German health insurance system, as well as the perception of patients with neurological conditions as particularly demanding for care teams. Examples of these challenges include communication impairments^15^ or strong wishes for autonomy.^18^

Addressing these collaborative and structural deficiencies could significantly improve access and quality of care for patients with neurological conditions at the end of life. Key steps include raising neurologists’ awareness of palliative care, lowering the barrier to referral, integrating education on neurological conditions into palliative care training and fostering more coordinated interprofessional care pathways.

## Conclusions

This study provides a current overview of hospice utilisation among patients with neurological diseases in Germany. Our findings underscore a gap in end-of-life care for patients with neurological conditions in Germany, mirroring international observations. While individuals with certain neurological conditions, like Parkinson’s disease, are under-represented in inpatient hospices, further research should explore the specific support requirements of these patients and their caregivers, as well as the structural limitations of existing care models. Understanding needs from various perspectives will reveal the true care gaps and inform future interventions.

Neurologists should recognise that their expertise remains invaluable for patients with neurological conditions, even at the very end of life. The vast majority of patients are cared for at their homes in the final months of life, and caregivers are challenged with their care. Strengthening outpatient initiatives that support severely ill patients with neurological conditions at home, together with a deeper engagement of neurologists in palliative care, will be crucial to improving quality of life and ensuring that neurological expertise benefits patients throughout their illness trajectory.

## Limitations

The findings of this point prevalence study on hospice usage for patients with neurological conditions must be considered in light of several limitations inherent to the online survey method. A primary limitation is the risk of self-selection bias, as the participating hospices are not a randomised sample and may not be fully representative of all German hospices. Although their data covers a substantial 37% of all inpatient hospice beds nationally, the study’s conclusions are not based on a probability sample. Furthermore, the survey’s reliance on self-reported data introduces the potential for recall bias or errors in reported patient counts and diagnostic detail, which may lead to imprecision in the descriptive analysis. This is evidenced by discrepancies found between the reported bed capacity in our survey and official statistics (eg, in Bavaria), suggesting either misreporting by individual hospices or unrecorded increases in capacity. Finally, while this study provides valuable point prevalence data, research in end-of-life care is also broadly limited by potential inaccuracies in official cause-of-death statistics, which are often restricted to a single underlying ICD-10 code and lack detailed clinical or sociodemographic context. Our study helps to provide a more nuanced picture of patient populations, but these broader data challenges remain a hurdle for the field.

## Supplementary material

10.1136/bmjno-2025-001404 online supplemental file 1

## Data Availability

Data are available upon reasonable request.
