# Supplementary material for: Mapping end-of-life care for patients with neurological conditions in German hospices: a point prevalence survey
Source: BMJ Neurol Open. 2026 Feb 25;8(1):e001404. doi: 10.1136/bmjno-2025-001404 (PMC12958885; doi:10.1136/bmjno-2025-001404)
Supplement: online supplemental file 1 [file bmjno-8-1-s001.docx]

**LimeSurvey Point Prevalence Study German Hospices**

**In which federal state is your facility located?** (Dropdown-Menu)

**What is your function within your facility?** [ ] Management [ ] Nursing Services Director [ ] Medical Director [ ] Other

**How many beds are available in your facility?** (Number)

**How many beds are currently occupied?** (Number)

**How many of your residents have a neurological disease as their primary diagnosis?** Please check the diagnoses that apply to your current residents and, if applicable, provide the number of affected individuals for each diagnosis.

[ ] Hypoxic brain injury [ ] ALS (Amyotrophic Lateral Sclerosis) [ ] Motor neuron disease [ ] Parkinson's and Parkinsonian syndromes (e.g., PSP, CBD, MSA) [ ] Multiple Sclerosis [ ] Primary brain tumors [ ] Other neurological disease

**How many of your residents have a pulmonary disease as their primary diagnosis?** Please check the diagnoses that apply to your current residents and, if applicable, provide the number of affected individuals for each diagnosis.

[ ] Interstitial lung disease (e.g., pulmonary fibrosis) [ ] COPD (Chronic Obstructive Pulmonary Disease) [ ] Other lung diseases

**How many of your residents have an oncological disease as their primary diagnosis (excluding primary brain tumors)?** Please enter numbers only.

**How many of your residents have a cardiovascular disease as their primary diagnosis?** Please enter numbers only.

**How many of your residents have another internal medical disease as their primary diagnosis?** Please enter numbers only.

**Do you have the option to consult a neurologist in your facility?** (Please check one)

[ ] No – no need [ ] No – it's not possible because no neurologist is available [ ] No – but it would be desirable as a goal [ ] Yes – it is possible to consult a neurologist on an ad hoc basis when needed [ ] Yes – a neurologist regularly visits the facility

**Do you have the option to consult a pulmonologist in your facility?**

[ ] No – no need [ ] No – it's not possible because no pulmonologist is available [ ] No – but it would be desirable as a goal [ ] Yes – it is possible to consult a pulmonologist on an ad hoc basis when needed [ ] Yes – a pulmonologist regularly visits the facility

**Do you offer spiritual care/chaplaincy services by qualified personnel to your residents?** (Please check one)

[ ] No – no need [ ] No – relatives can organize this independently [ ] No – but it would be desirable as a goal [ ] Yes – there is a formal cooperation with a church [ ] Yes – a spiritual care provider regularly visits the facility [ ] Yes – we have contact with spiritual care providers whom we can request when needed [ ] Yes – if these are integrated via SAPV (Specialized Outpatient Palliative Care)

**Would you be interested in telemedicine consultation services in the form of video consultations?**

[ ] Yes [ ] No
